# Harmonized multi-cohort analysis of oral microbiome development during early lifetime

**DOI:** 10.1080/20002297.2026.2728748

**Published:** 2026-09-11

**Authors:** Do Yeon Won, Si Yeong Kim, Seon Mi Kim, Hyewon Shin, Hee Sam Na, Jin Chung

**Affiliations:** a Department of Pediatric Dentistry, School of Dentistry, Chonnam National University, Gwangju, Republic of Korea; b Department of Oral Microbiology, School of Dentistry, Chonnam National University, Gwangju, South Korea; c Department of Oral Microbiology, School of Medicne, Chonnam National University, Gwangju, Republic of Korea

**Keywords:** Oral microbiome, early childhood, longitudinal, 16S rRNA, multi-cohort analysis, alpha/beta diversity, temporal clustering, succession, pediatric dentistry

## Abstract

**Background:**

Early-life assembly of the oral microbiome may influence oral and systemic health, yet developmental patterns are difficult to compare across studies because of differences in sampling and analytical workflows. We conducted a harmonized cross-cohort re-analysis of longitudinal pediatric 16S rRNA datasets spanning birth to early childhood.

**Methods:**

Five publicly available longitudinal cohorts from birth to seven years were curated and independently reprocessed using QIIME 2 and R. Taxonomy was assigned using the Human Oral Microbiome Database (HOMD). Alpha and beta diversity, taxonomic composition, and species-level temporal dynamics were evaluated. Age was harmonized to months and summarized into three developmental intervals: birth–12 months, 12–36 months, and >36 months. Temporal clustering used fuzzy c-means clustering (TCseq).

**Results:**

Alpha diversity increased rapidly during the first year, plateaued between one and three years, and increased gradually thereafter. Beta diversity showed the greatest compositional shifts during infancy, followed by relative stabilization. Taxonomic profiling revealed reproducible succession from *Streptococcus* and *Staphylococcus* to *Haemophilus*, *Neisseria*, and *Veillonella*, followed by *Prevotella* and *Campylobacter* after three years. Temporal clustering identified three developmental groups corresponding to early, transitional, and later-expanding taxa.

**Conclusions:**

This harmonized analysis of five longitudinal cohorts demonstrates a reproducible three-phase trajectory of oral microbiome maturation during early childhood. The most pronounced microbial restructuring occurred during infancy, followed by community stabilization and gradual maturation. These findings provide a framework for understanding age-associated microbial succession and identifying developmental windows for preventive interventions in pediatric oral health.

## Introduction

Early-life development of the oral microbiome is increasingly recognised as an important factor influencing oral and systemic health across the lifespan [1,2]. The first colonisers assemble at birth and are shaped by delivery mode, feeding practices, antibiotic exposures, and environmental conditions, after which the community undergoes rapid, non-linear remodelling through infancy and early childhood [2,3]. These early successional dynamics contribute to maturation of mucosal immunity and stabilisation of ecological networks; conversely, disruptions have been associated with elevated risks of dental caries and other adverse outcomes later in life [1–3].

The early oral habitat undergoes structured change from birth through the preschool years. In the neonatal period, the mouth is dominated by moist, shedding mucosa with low biomass; during the first year, salivary glands mature, flow and buffering capacity increase, and secretory factors such as mucins and immunoglobulins rise, collectively shaping adhesion, shear, and pH microenvironments on soft tissues. Beginning around six months, eruption of primary teeth introduces non-shedding enamel surfaces and nascent gingival sulci, adding oxygen and redox gradients and new attachment sites for biofilm assembly [4,5].

Microbial succession tracks these habitat shifts. Early infancy commonly features facultative pioneers such as *Streptococcus* (including *S. salivarius*), *Gemella*, and *Rothia*, with *Veillonella* exploiting lactate released by saccharolytic streptococci; this fosters localised oxygen depletion and co-adhesion scaffolds for later colonisers [6,7]. As teeth erupt and diet diversifies during the second year, communities incorporate more *Neisseria* and *Haemophilus* and subsequently broaden to include anaerobic guilds such as *Prevotella*, *Campylobacter*, and *Fusobacterium* as maturing plaque structures stabilise on non-shedding tooth surfaces [8–10]. However, mechanistic insight into these early interactions has historically been derived largely from culture-based isolation and in vitro coaggregation studies, whereas age-resolved, NGS-based longitudinal profiling in infancy and toddlerhood remains comparatively sparse and difficult to synthesise across studies because of methodological heterogeneity in sampling sites and workflows [6,7,11–13].

Despite these advances, direct comparisons across studies remain challenging because of heterogeneity in sampling sites and schedules, targeted 16S regions, and analytic workflows; such variability can obscure shared developmental signals and complicate generalisation [12].

To address these gaps, we curated and harmonised five publicly available longitudinal 16S rRNA datasets on paediatric oral microbiome development spanning birth through seven years (PRJDB17623, PRJEB35824, PRJEB66628, PRJEB60183, PRJNA803343) and applied a single, standardised processing and analysis pipeline across cohorts [10,14–17].

Within this integrative framework, we categorised temporal community dynamics into three age-based clusters—birth to 12 months, 12 to 36 months, and after 36 months—to delineate reproducible developmental trajectories and to pinpoint critical windows for preventive intervention.

## Materials and methods

### Search criteria

Studies were collected by using keyword searches on PubMed. Search terms used aimed to firstly select for microbiome and NGS studies, and then, additional search terms were added using the ‘AND’ function to further narrow search results down to those performed on the oral microbiome, specifically related to early microbiome development. All PubMed search results were exported to EndNote and upon exportation study titles and abstracts were screened for relevance and shortlisted. Studies were excluded that were not related to the oral microbiome 16 s rRNA, had no data accession number, were not mappable to individual samples, and had no additional metadata like age, sex, and sampling site of the individuals under study. We also excluded studies with data ‘available upon reasonable request’, *in vitro* studies, transcriptomic studies and data deposited in other repositories other than the National Centre for Biotechnology Information (NCBI) or European Nucleotide Archive (ENA) database.

### Data retrieval and processing

Raw sequence data were downloaded from the ENA database before quality control protocols were carried out using a standardised pipeline in Qiime2 [18]. Paired-end reads were demultiplexed with q2-demux and quality filtered, denoised, dereplicated, and screened for chimeras with q2-dada2 to generate amplicon sequence variants (ASVs). Representative sequences were assigned taxonomy against the Human Oral Microbiome Database (HOMD) using a pretrained naive Bayes classification framework. Feature tables and study metadata were then imported into R for within-cohort analysis [19,20].

### Cross-cohort analytical framework

Because the five included longitudinal cohorts differed substantially in sampling schedules, age distributions, geographic populations, sequencing protocols, and targeted 16S rRNA variable regions, direct pooling of individual samples into a single longitudinal analytical model was not considered appropriate. In particular, sampling time points were not consistently aligned across cohorts, and direct harmonisation would have required aggregation, interpolation, or imputation of age-specific observations, potentially introducing additional assumptions and obscuring study-specific biological variation. We therefore adopted a two-tier cross-cohort analytical framework. PRJDB17623, which provided the most detailed longitudinal sampling across early childhood, was used as the representative cohort for detailed visualisation of age-associated microbial trajectories. The remaining four cohorts were analysed independently and used as replication cohorts to evaluate the reproducibility of the principal directional patterns observed in the representative dataset, including changes in alpha diversity, community composition, taxonomic succession, and temporal microbial trajectories. Cross-cohort conclusions were therefore based on recurring patterns observed across independent datasets rather than on pooled sample-level estimates.

To reduce additional technical variation, microbiome analyses were performed independently within each cohort, so that longitudinal changes were evaluated within the same study-specific sampling and sequencing framework. Relative-abundance-based beta-diversity and temporal clustering analyses were used primarily as descriptive approaches to characterise within-cohort developmental trajectories rather than to infer absolute changes in individual taxa or direct ecological interactions. We acknowledge that this study-specific analytical strategy does not eliminate the inherent compositional nature of relative-abundance data. Accordingly, interpretation emphasised broad temporal patterns and taxonomic trajectories that were reproducibly observed across independent cohorts, rather than isolated changes identified within a single dataset.

### Microbiome analyses

We calculated sample-based metrics upon the common alpha diversity metrics Chao and Shannon within a given sample [21,22]. Alpha diversity metrics aim to apply an individual metric to each individual sample's microbiome. Chao1 estimates overall species richness while the Shannon index increases with both richness and evenness of the community. Analyses were performed on a per-study basis followed by on all studies collectively. The phyloseq package (v1.30.0) was used to calculate alpha diversity [23]. Beta-diversity indices were employed to infer dissimilarity between samples. Beta-diversity distances were also calculated in phyloseq. Ggplot2 was used for visualisation and combining of plots within R. Permutational multivariate analysis of variance (PERMANOVA) was performed on the beta diversity distance matrix which was assessed for statistical significance using the ADONIS function within the vegan package. To characterise the microbial variation pattern depending on age, the expression mode clustering was analysed and visualised using the time course sequencing (‘TCseq’) package (version 1.27.0).

### Statistical analysis

All statistical analyses and graph production were performed using the appropriate functions in R (4.1.2) unless stated otherwise. Statistical significance was determined when *p* < 0.05.

## Results

### Study overview and participant information

We finally selected five studies and performed a multi-cohort analysis by combining five publicly available 16S rRNA gene sequencing datasets (PRJDB17623, PRJEB35824, PRJEB66628, PRJEB60183, and PRJNA803343), all targeting the oral microbiome during early life. Across cohorts, the aggregate sample size was *n* = 859 (54, 189, 90, 260, and 266, respectively), with sampling from as early as Day 2/Week 1 after birth up to 84 months. Sex metadata were available in three cohorts (PRJDB17623, PRJEB35824, PRJEB60183). Each dataset was analysed independently to characterise age-associated changes in oral microbiome composition, and the results were subsequently integrated to infer developmental patterns across early childhood. Table 1 summarises the sample characteristics across the included datasets.

**Table 1.** Study design and characteristics of 5 publications analysed.

Publication	Location	Sample number	Age	Gender	Target Variable region	Data Accession
Sato, 2023	Japan	54	1 W, 1 M, 3 M, 6 M, 9 M, 12 M, 18 M, 24 M, 30 M, 36 M, 42 M, 48 M, 60 M	Male: 27, Female: 27 (50%/50%)	V1–V2	PRJDB17623
Blostein, 2022	USA	189	D2, M3, M18, M36, M60	Male: 97, Female: 92 (51%/49%)	V3–V4	PRJEB35824
Dzidic, 2018	Sweden	90	3 M, 6 M, 12 M, 24 M, 7Y	−	V3–V4	PRJEB66628
Eriksen, 2024	Sweden	260	2Y, 3Y, 5Y	Male: 126, Female: 134 (48%/52%)	V3–V4	PRJEB60183
Kahharova, 2023	USA	266	~1Y, ~2.5Y, ~4Y	−	V4	PRJNA803343

Age indicates sampling time points reported in each study. D, days; W, weeks; M, months; Y, years. Target variable region refers to the 16S rRNA gene hypervariable region sequenced in each cohort. Sample number represents the total number of samples included in the original study. Gender information is reported as provided in the source publications; ‘–’ indicates data not reported.

### Microbial diversity changes during early growth

**Figure 1.**
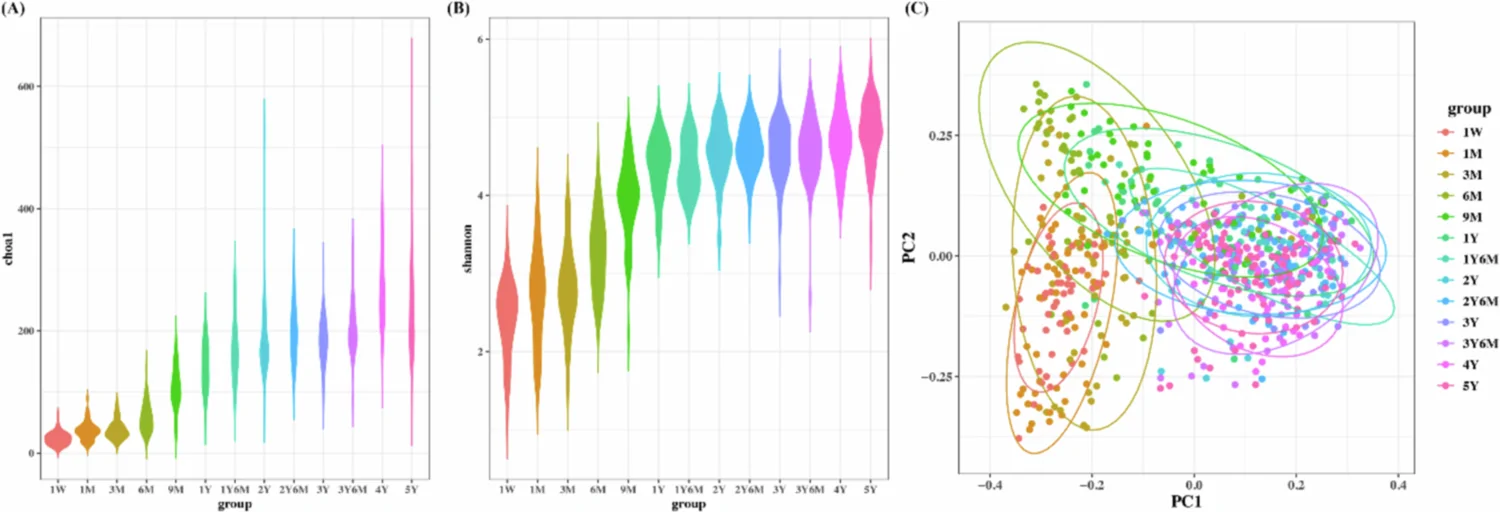
Age-associated changes in oral microbiome diversity during early childhood. Age-associated changes in oral microbiome diversity in the representative longitudinal cohort PRJDB17623. (A) Chao1 richness and (B) Shannon diversity indices showing progressive increases in alpha diversity across early childhood. Both indices increased rapidly during the first year of life, followed by relative stabilisation between 1 and 3 years and gradual increases thereafter. (C) Principal coordinates analysis (PCoA) based on Bray–Curtis dissimilarity demonstrating age-associated shifts in community composition. Samples collected during infancy (1 week–12 months) were compositionally distinct and more dispersed, whereas samples obtained after 2 years of age clustered more closely, indicating progressive stabilisation of the oral microbiome with age.

The alpha diversity of the oral microbiota was estimated using the Chao1 and Shannon indices. For representative presentation, the PRJDB17623 results were visualised (Figure 1A,B). This dataset included samples from children aged 1 week (1 W) to 5 years (5Y), allowing detailed observation of diversity changes during early life. The Chao1 index (richness) steadily increased with age, and the Shannon index (richness + evenness) also increased over time. In particular, both indices rose sharply during the first year of life, followed by a period of relative stability between 1 and 3 years (1Y–3Y). After 3 years (3Y), diversity continued to rise gradually, approaching a more stable state by 5 years (5Y). Similar trends were observed in other datasets: PRJEB66628 and PRJNA803343 showed comparable age-associated increases in both indices. Although PRJEB35824 and PRJEB60183 included fewer sampling timepoints and exhibited relatively flatter trajectories, the overall trend of increasing alpha diversity with age remained consistent (Fig. S1). Together, these patterns delineate a 0–12 months surge, a 12–36 months plateau, and over 36 months gradual maturation, which we use as practical windows in downstream summaries.

The beta diversity of the oral microbiota was evaluated using Bray–Curtis distances and visualised via principal coordinates analysis (PCoA). In PRJDB17623, age-associated clustering patterns were observed (Figure 1C): samples from 1W–1Y were more dispersed and compositionally distinct, whereas those from 2Y–5Y were more tightly grouped. PERMANOVA confirmed significant compositional differences between early age groups, particularly before versus after 1 year of age; by contrast, no significant differences were detected among 2–5 years. A complementary analysis focusing on 12 M, 30 M, and 48 M in PRJNA803343 showed a clear separation between 12 M and 30 M, with 30 M and 48 M largely overlapping (Fig. S2). These findings indicate that the largest structural shift occurs before/around 12 months, with comparatively modest restructuring thereafter.

### Taxonomic composition across ages

**Figure 2.**
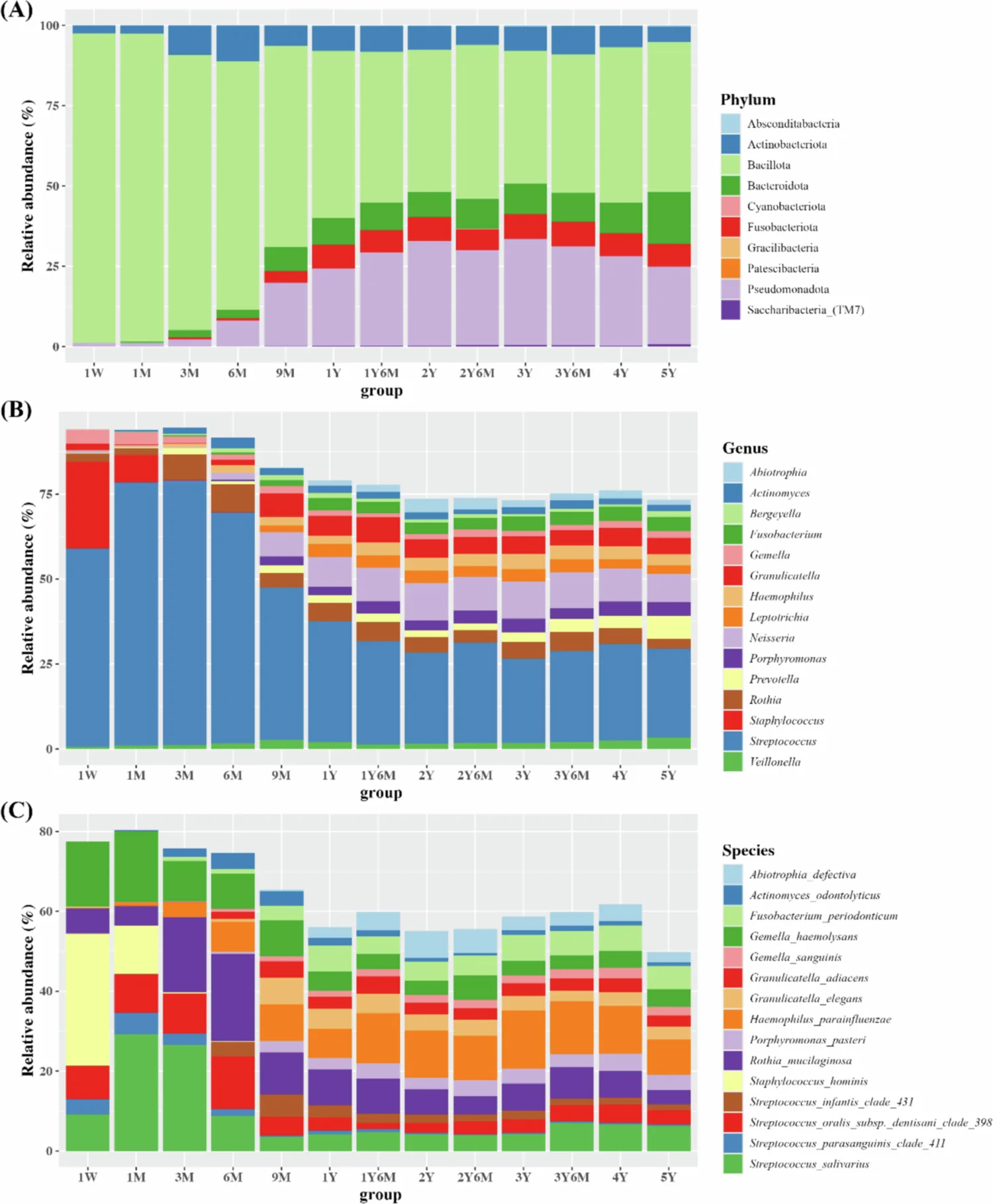
Taxonomic succession of the oral microbiome across developmental stages. Age-related taxonomic composition of the oral microbiome in the representative cohort PRJDB17623. (A) Relative abundance of dominant bacterial phyla across age groups. (B) Relative abundance of major bacterial genera showing progressive shifts during development. Early communities were dominated by *Streptococcus* and *Staphylococcus*, whereas *Haemophilus*, *Neisseria*, *Prevotella*, and *Porphyromonas* increased with age. (C) Relative abundance of representative bacterial species demonstrating distinct developmental trajectories from infancy through early childhood. Together, these data illustrate a continuous process of microbial succession accompanying oral habitat maturation.

Following taxonomic assignment, the average relative abundance of each phylum was plotted. In PRJDB17623, the dominant phyla during early childhood were Bacillota, Pseudomonadota, Bacteroidota, Fusobacteriota, and Actinobacteriota. Bacillota accounted for over 1% from 1 W to 1Y and maintained a similarly high proportion up to 5Y. Pseudomonadota increased from 6 M and remained stable until 3Y, after which its relative abundance decreased. Fusobacteriota and Bacteroidota emerged around ~9M and persisted thereafter (Figure 2A). At the genus level, *Streptococcus* and *Staphylococcus* predominated from 1 W to 1Y; although slightly declining after 1Y, both remained stable through 5Y. *Haemophilus* and *Neisseria* increased from 3 M and 6 M, respectively, peaked at 2–3Y, and then decreased. *Porphyromona*s and *Prevotella* began increasing from 9 M, remained stable during early childhood, and rose further after 2Y6M and 3Y, respectively (Figure 2B). These age-related genus-level shifts were consistently observed across the other four datasets (Fig. S3). At the species level, distinct transitions were evident (Figure 2C): *Streptococcus salivarius* was high at 1 M and 3 M; *Streptococcus oralis* subsp. *dentisani* remained elevated from 1 W to 6 M; *Streptococcus infantis* increased from 6 M and stabilised; *Staphylococcus hominis* peaked at 1 W and remained noticeable until 1 M; *Gemella haemolysans* was high from 1 W to 9 M and then stabilised, a trend mirrored in PRJEB35824 (Fig. S3A). *Gemella sanguinis* rose from 6 M and stabilised; *Granulicatella elegans* increased from 9 M, remained high until 3Y, then declined; *Granulicatella adiacens* rose from 6 M and stabilised, paralleling PRJEB66628 (Fig. S3B). *Fusobacterium periodonticum* appeared around 3 M and persisted after 9 M. Across cohorts, these successional patterns collectively reflect early colonisation, toddler-age maintenance, and later maturation phases.

### Temporal clustering of microbial communities

**Figure 3.**
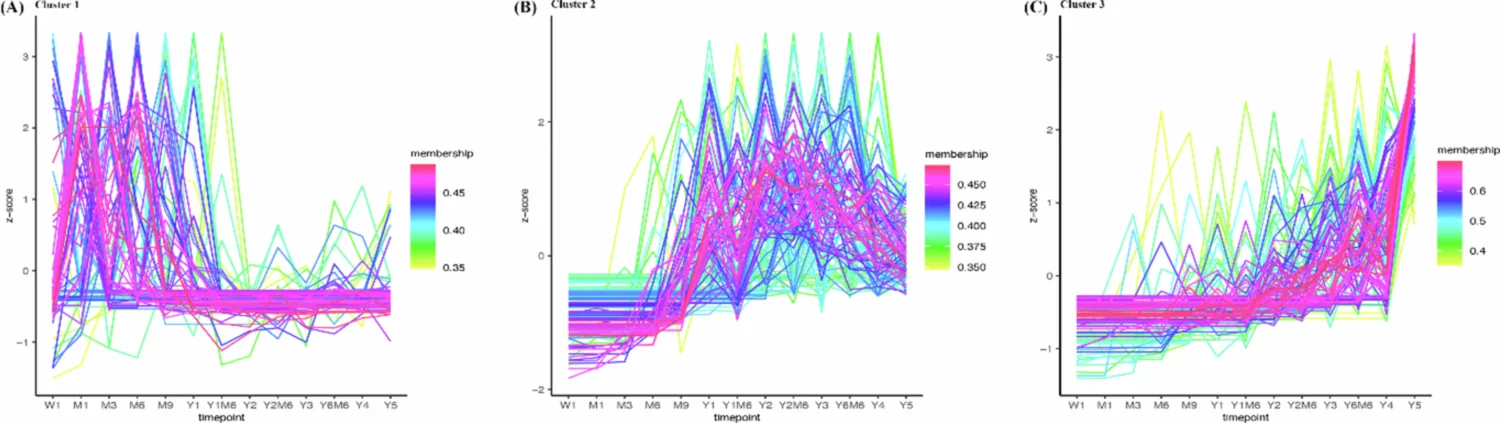
Temporal clustering of oral microbial species during early-life development. Temporal clustering of species-level microbial trajectories using fuzzy c-means clustering. Species were grouped according to longitudinal abundance patterns across developmental stages. (A) Cluster 1 comprises early colonisers that peak during infancy and decline thereafter. (B) Cluster 2 includes taxa that increase during late infancy and remain relatively abundant throughout toddlerhood. (C) Cluster 3 consists of taxa that gradually expand during later childhood. The reproducible identification of these three temporal patterns supports the existence of distinct developmental phases during oral microbiome maturation.

Temporal clustering at the species level captured developmental dynamics across early childhood (Figure 3). Using fuzzy c-means on time-normalised abundances, three temporal clusters were identified in PRJDB17623, a structure replicated across the other four datasets (Fig. S4). Cluster 1 showed an early peak (1M–1Y) followed by decline; Cluster 2 increased rapidly by 6–9M, maintained relatively high levels between 1Y and 3Y, then declined; Cluster 3 remained low during infancy but increased progressively from 2–3Y, reaching the highest levels at 4–5Y. These temporal clusters correspond to the birth-to-12-month, 12-to-36-month, and post-36-month windows used to summarise alpha/beta diversity trends.

### Cluster-specific species abundance

**Figure 4.**
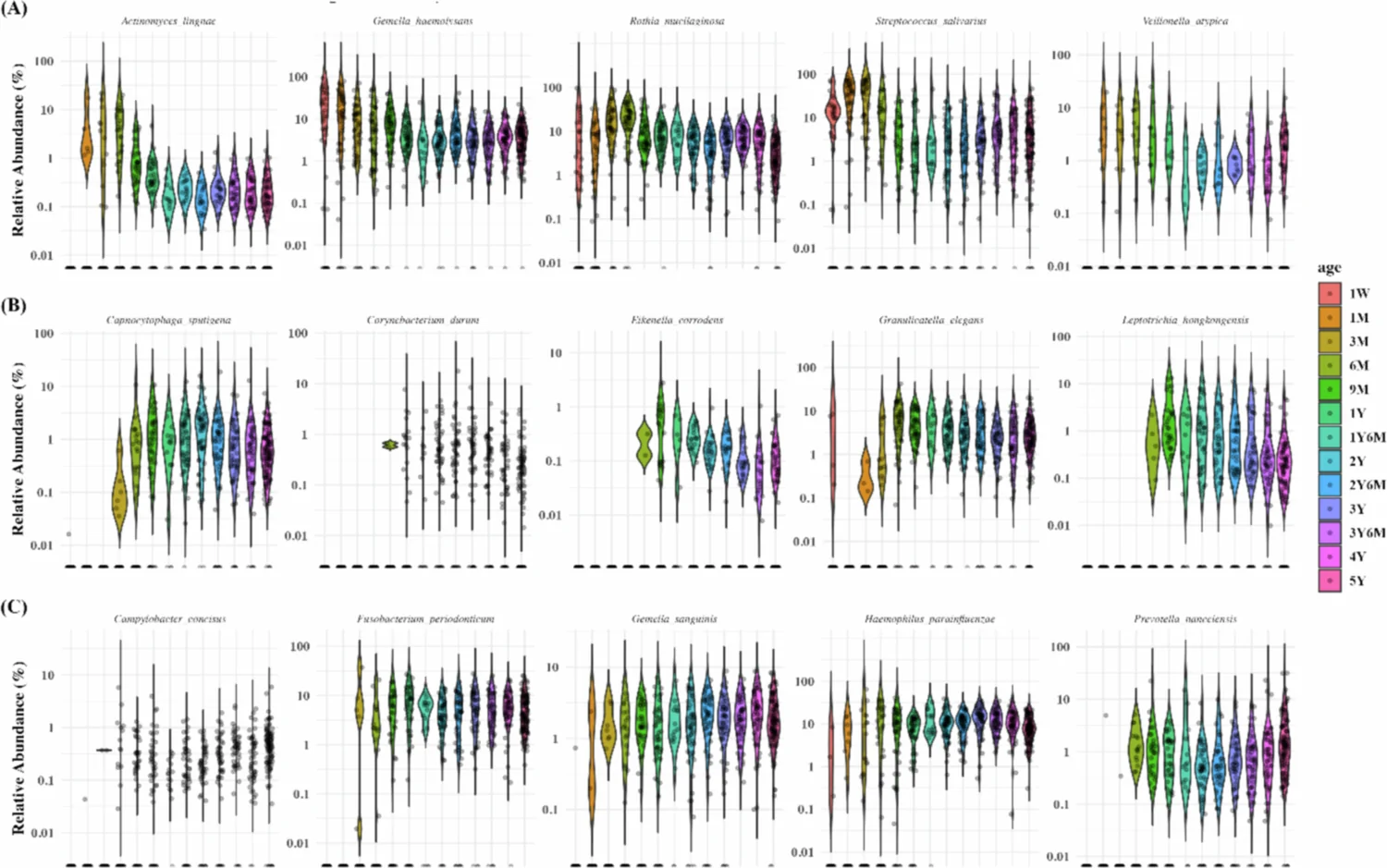
Representative species associated with developmental microbial clusters. Relative abundance distributions of representative species belonging to each temporal cluster identified by fuzzy c-means analysis. (A) Cluster 1 species, including *Actinomyces lingnae*, *Gemella haemolysans*, *Rothia mucilaginosa*, *Streptococcus salivarius*, and *Veillonella atypica*, were enriched during early infancy and declined with age. (B) Cluster 2 species, including *Capnocytophaga sputigena*, *Corynebacterium durum*, *Eikenella corrodens*, *Granulicatella elegans*, and *Leptotrichia hongkongensis*, increased during late infancy and remained relatively stable throughout toddlerhood. (C) Cluster 3 species, including *Campylobacter concisus*, *Fusobacterium periodonticum*, *Gemella sanguinis*, *Haemophilus parainfluenzae*, and *Prevotella nanceiensis*, expanded during later childhood. These taxa collectively represent the sequential stages of oral microbiome maturation from infancy to early childhood.

To determine common taxa within clusters, per-dataset memberships were combined and the relative abundance of taxa shared across cohorts was visualised for PRJDB17623 as a representative case (Figure 4). Cluster 1 included early colonisers such as *Actinomyces lingnae*, *G. haemolysans, Rothia mucilaginosa*, and *Veillonella atypica*, which showed high relative abundance during early infancy and gradually decreased thereafter (Figure 4A). Cluster 2 comprised species such as *Capnocytophaga sputigena*, *Corynebacterium durum*, *Eikenella corrodens*, *G. elegans*, and *Leptotrichia hongkongensis*, which exhibited a rapid increase during the first year of life, maintained relatively high abundance during early childhood, and declined thereafter (Figure 4B). Cluster 3 included *Campylobacter concisus*, *G. sanguinis*, *H. parainfluenzae*, and *Prevotella nanceiensis*, which increased after 2–3Y and peaked by 4–5Y (Figure 4C). The recurrence of these taxa across multiple cohorts supports a convergent three-phase maturation despite differences in variable regions and sampling density.

## Discussion

Understanding how the oral microbiome develops during early life is important because microbial colonisation and maturation during infancy and early childhood may influence long-term oral health outcomes [1,2]. However, comparisons across studies have remained difficult because longitudinal paediatric microbiome datasets differ considerably in sampling schedules, sequencing targets, and analytical workflows [11–13]. In the present study, we harmonised and reprocessed five independent longitudinal 16S rRNA cohorts using a single analytical pipeline [10,14–17]. This approach was selected because a narrative summary would retain study-specific differences in preprocessing and taxonomic assignment; reference-database selection itself can materially alter taxonomic classification and biomarker recovery [24]. The revised analysis identifies recurring age-associated patterns while avoiding pooled ASV-level inference across technically heterogeneous cohorts. (Figures 1–4).

Across cohorts, alpha diversity increased markedly during the first year of life and became relatively stable between one and three years of age, followed by a more gradual increase thereafter (Figure 1A,B; Fig. S1). This pattern is compatible with rapid ecological assembly during infancy followed by slower consolidation as salivary function matures, teeth erupt, and dietary substrates diversify. However, the magnitude and exact timing differed among cohorts, and the three age intervals used here should be regarded as descriptive summaries rather than fixed developmental stages.

Similarly, beta diversity analyses demonstrated that microbial communities before one year of age were compositionally more dispersed and distinct, whereas samples collected after approximately two years clustered more closely together (Figure 1C; Fig. S2). These findings suggest that infancy represents a period of rapid microbial assembly and restructuring, while toddlerhood corresponds to a comparatively more stable phase of community consolidation. Similar age-associated increases in oral microbial diversity during infancy and gradual stabilisation during early childhood have also been reported in previous longitudinal studies [3,8–10].

The taxonomic succession patterns identified in this study were also highly consistent across cohorts despite differences in sampling density and sequencing regions. *Streptococcus*-dominant assemblages with *Veillonella* and *Gemella* were prominent during infancy; *Neisseria, Haemophilus, Capnocytophaga, Granulicatella,* and *Leptotrichia* became more prominent during toddlerhood; and *Prevotella* and *Campylobacter* increased later (Figure 2; Figure 4; Fig. S3) [5–10,25–27]. These sequential microbial shifts likely reflect major developmental and environmental transitions occurring during early life, including maturation of salivary function, eruption of primary teeth, diversification of diet, and establishment of oral hygiene habits [4,5].

Temporal clustering identified three reproducible phases of early oral microbiome development (Figure 3; Fig. S4): taxa that peaked during infancy and subsequently declined, taxa that increased in late infancy and remained stable through toddlerhood, and taxa that expanded after approximately 2–3 years of age. These patterns were observed across independent cohorts despite differences in sampling schedules and 16S rRNA variable regions, indicating shared developmental transitions. The most pronounced compositional changes occurred between approximately 6 months and 3 years, coinciding with primary tooth eruption, dietary diversification, and the establishment of oral hygiene practices [5,8–10]. This period may therefore represent an important window for future studies of microbiome-informed preventive approaches.

From a clinical perspective, the developmental period identified in this study coincides with the introduction of dietary and oral hygiene practices that are already recognised as important for maintaining oral health. Measures such as limiting frequent sugar exposure and sugar-sweetened beverages, together with initiating age-appropriate oral hygiene, remain relevant during infancy and toddlerhood [15,17,28,29]. However, because the included cohorts did not evaluate preventive interventions or clinical outcomes, our findings do not establish that microbiome-directed intervention during this period produces greater long-term benefit. Rather, they provide a descriptive framework for understanding age-related microbial maturation in early childhood.

This framework may also provide useful context for future studies of early childhood caries (ECC), but no direct association with ECC can be inferred from the present analysis. ECC is a common chronic oral disease in preschool children and is strongly associated with frequent sugar exposure and prolonged acidification of dental biofilms [29]. Previous studies have reported enrichment of acidogenic and acid-tolerant taxa, including *Streptococcus mutans, Scardovia wiggsiae,* and *Lactobacillus spp.,* in ECC-associated communities [30–32]. Although the most pronounced microbial restructuring in our analysis occurred before approximately three years of age, the included cohorts did not longitudinally assess caries development, dietary exposure, or oral hygiene behaviour. Therefore, whether alterations in normal microbial maturation contribute to subsequent caries susceptibility remains to be determined in prospective studies incorporating detailed clinical and behavioural data.

Importantly, this study was designed to identify broad developmental patterns that were reproducible across independent cohorts rather than to resolve narrow, month-specific fluctuations. The included longitudinal datasets differed substantially in sampling intervals, visit timing, participant numbers at individual ages, and geographic origin. We therefore summarised microbiome trajectories using broader developmental windows instead of directly comparing every month across studies. Although this approach limits fine-scale temporal interpretation, it reduces the influence of irregular sampling schedules and provides a practical framework for identifying shared developmental transitions across heterogeneous cohorts.

Comparison with later life provides useful context, although direct continuity cannot be assumed. In a recent cross-sectional study of supragingival plaque from 533 Korean participants, alpha diversity was lower in children and young adults and higher in middle-aged and older adults; younger groups had greater relative abundance of early-associated genera such as *Streptococcus, Veillonella, Neisseria,* and *Haemophilus,* whereas *Porphyromonas, Prevotella, Fusobacterium,* and *Treponema* were more prominent in older groups [33]. These observations are broadly compatible with progressive diversification and increasing representation of anaerobic taxa after childhood. These findings suggest that early-life oral microbiome maturation may represent the beginning of a broader age-associated trajectory characterised by increasing diversity and a gradual shift toward more anaerobe-rich communities. However, because the adult comparison was cross-sectional and involved supragingival plaque, longitudinal studies are required to confirm continuity from childhood into later life.

This study has several limitations. First, substantial methodological heterogeneity remained among the five included cohorts, including differences in longitudinal sampling schedules, ages at specimen collection, geographic populations, sequencing protocols, and targeted 16S rRNA variable regions. One cohort targeted the V1–V2 region of the 16S rRNA gene, three targeted V3–V4, and one targeted V4. Because different variable regions differ in primer coverage and taxonomic discriminatory capacity, particularly for closely related oral taxa, some between-study variation may reflect technical differences rather than biological variation alone [11–13]. In addition, the sampling time points and visit intervals were not directly aligned across cohorts. Consequently, formal pooling of the individual-level data or quantitative meta-analysis of age-associated effects would have required substantial aggregation, interpolation, or other assumptions to harmonise longitudinal time points across studies. We therefore analysed each cohort independently and focused on broad developmental patterns that were reproducible across datasets rather than estimating pooled effect sizes or formal measures of between-study heterogeneity. Although this strategy allowed us to identify recurring developmental trajectories while preserving study-specific characteristics, it limits direct quantitative comparison among cohorts. Future prospective multi-centre studies using harmonised sampling schedules, sequencing protocols, and metadata collection will be required to enable pooled longitudinal modelling and formal quantitative synthesis.

Second, several analyses in the present study were based on relative-abundance data. Because microbiome data are compositional, an apparent change in the relative abundance of one taxon may partly reflect changes in other members of the microbial community rather than an absolute change in that taxon itself. This issue is particularly relevant to analyses of community dissimilarity and temporal clustering and should be considered when interpreting age-associated compositional shifts. Furthermore, because the present study relied on short-read 16S rRNA sequencing of different partial variable regions, species-level assignments should be interpreted as putative rather than definitive. Closely related oral species, particularly within Streptococcus, may share highly similar sequences within the amplified regions and therefore cannot always be reliably distinguished. Future studies incorporating absolute microbial quantification and compositional-data-aware approaches, together with metagenomics and metabolomics, will be important for confirming the taxonomic and functional significance of the developmental transitions identified here [1,2,34–39].

Third, all included datasets were derived from observational longitudinal studies, and clinical, developmental, and behavioural covariates were incompletely and inconsistently recorded. Feeding mode, timing of weaning, introduction of solid foods, dietary sugar exposure, antibiotic exposure, tooth-eruption status, fluoride exposure, and oral-hygiene practices were unavailable or not consistently reported across cohorts. These factors may influence oral microbial maturation by altering nutrient availability, surface accessibility, salivary conditions, and biofilm formation [15,17,28,29]. Their absence prevented harmonised adjustment and limits causal interpretation of the observed age-associated changes. The detected trajectories should therefore be regarded as patterns of chronological maturation rather than effects attributable to specific dietary, developmental, or behavioural exposures.

Finally, an additional methodological limitation concerns the compositional nature of microbiome sequencing data. Several analyses in the present study, including beta-diversity assessment and fuzzy c-means temporal clustering, were performed using relative-abundance information without explicit log-ratio transformation. Because an increase or decrease in the relative abundance of one taxon is necessarily dependent on the abundances of other community members, these analyses cannot distinguish relative compositional changes from absolute changes in microbial abundance. Accordingly, the temporal clusters identified here should be interpreted as descriptive patterns of relative community succession rather than as evidence of absolute expansion or depletion of individual taxa or direct ecological associations. We attempted to reduce additional technical heterogeneity by analysing each longitudinal cohort independently and by emphasising developmental patterns that recurred across independent datasets; however, this strategy does not eliminate the fundamental constraint of compositional data. Future studies employing prospectively harmonised sampling designs, compositional-data approaches such as log-ratio transformation and Aitchison distance, and, where feasible, absolute microbial quantification will be important for validating these developmental trajectories.

In conclusion, this cross-cohort multi-cohort analysis identified a reproducible three-phase pattern of oral microbiome development during early life, consisting of rapid early remodelling during infancy, transitional stabilisation during toddlerhood, and gradual maturation thereafter (Figures 1–4). Importantly, the largest microbial changes occurred before approximately three years of age, suggesting that infancy and early toddlerhood may represent important developmental windows for microbiome-directed preventive interventions. These findings provide a developmental framework for understanding early-life oral microbiome maturation and may help guide future preventive approaches in paediatric oral health research.

## Supplementary Material

Supplementary_Figures.docx Supplementary Material
