# Supplementary material for: Harmonized multi-cohort analysis of oral microbiome development during early lifetime
Source: J Oral Microbiol. 2026 Sep 11;18(1):2728748. doi: 10.1080/20002297.2026.2728748 (PMC13573508; doi:10.1080/20002297.2026.2728748)
Supplement: Supplementary file 1 [file ZJOM_A_2728748_SM6986.docx]

Supplementary Figure 1

**Age-associated changes in alpha diversity across independent longitudinal cohorts**

Alpha diversity dynamics across four independent longitudinal oral microbiome cohorts. Chao1 richness and Shannon diversity indices were calculated for (A) PRJDB17623, (B) PRJEB35824, (C) PRJEB66628, and (D) PRJNA803343. Across cohorts, alpha diversity generally increased with age, with the most pronounced increases occurring during the first year of life, followed by relative stabilization during toddlerhood and gradual maturation thereafter. Despite differences in sampling schedules and cohort composition, overall diversity trajectories were broadly consistent among studies.

Supplementary Figure 2

**Beta diversity analyses of age-associated microbial community shifts**

Principal coordinates analysis (PCoA) based on Bray–Curtis dissimilarity demonstrating age-associated changes in oral microbiome composition across independent cohorts. (A) PRJDB17623, (B) PRJEB35824, (C) PRJEB66628, and (D) PRJNA803343. Early-life samples displayed greater dispersion and compositional variability, whereas samples collected at later ages clustered more closely. Consistent age-related community transitions were observed across datasets, supporting progressive stabilization of the oral microbiome during childhood.

Supplementary Figure 3

**Taxonomic composition of the oral microbiome across developmental stages**

Relative abundance of dominant bacterial taxa across age groups in independent longitudinal cohorts. Upper panels show phylum-level composition, whereas lower panels show genus-level composition for (A) PRJDB17623, (B) PRJEB35824, (C) PRJEB66628, and (D) PRJNA803343. Across cohorts, early communities were enriched in *Streptococcus* and other pioneer taxa, whereas age-associated increases in *Haemophilus*, *Neisseria*, *Prevotella*, and other later colonizers were consistently observed. These findings support a conserved pattern of microbial succession during oral microbiome maturation.

Supplementary Figure 4

**Temporal clustering of microbial trajectories across independent cohorts**

Fuzzy c-means clustering of species-level temporal abundance trajectories across longitudinal cohorts. Temporal clusters were generated for (A) PRJDB17623, (B) PRJEB35824, (C) PRJEB66628, and (D) PRJNA803343. Each line represents the standardized abundance trajectory (z-score) of an individual species, with color intensity corresponding to cluster membership values. Three reproducible temporal patterns were consistently identified across cohorts: an early colonizer cluster enriched during infancy, a transitional cluster predominating during toddlerhood, and a later-expanding cluster that increased during later childhood. These reproducible clustering patterns support the existence of conserved developmental phases during early-life oral microbiome maturation.
